# Opposing Effects of PI3K/Akt and Smad-Dependent Signaling Pathways in NAG-1-Induced Glioblastoma Cell Apoptosis

**DOI:** 10.1371/journal.pone.0096283

**Published:** 2014-04-23

**Authors:** Zhiguo Zhang, Lin Wu, Julei Wang, Gang Li, Dayun Feng, Bin Zhang, Lihong Li, Jiandong Yang, Lianting Ma, Huaizhou Qin

**Affiliations:** 1 Department of Neurosurgery and Institute for Functional Brain Disorders, Tangdu Hospital, The Fourth Military Medical University, Xi’an, China; 2 Department of Biochemistry and Molecular Biology, State Key Laboratory of Cancer Biology, The Fourth Military Medical University, Xi’an, China; 3 Department of Hepatobiliary Surgery, Xijing Hospital, The Fourth Military Medical University, Xi’an, China; 4 Postdoctoral research station of Neurosurgery, Wuhan General Hospital of Guangzhou Command, PLA, Wuhan, China; University of Birmingham, United Kingdom

## Abstract

Nonsteroidal anti-inflammatory drug (NSAID) activated gene-1 (NAG-1) is a divergent member of the transforming growth factor-beta (TGF-β) superfamily. NAG-1 plays remarkable multifunctional roles in controlling diverse physiological and pathological processes including cancer. Like other TGF-β family members, NAG-1 can play dual roles during cancer development and progression by negatively or positively modulating cancer cell behaviors. In glioblastoma brain tumors, NAG-1 appears to act as a tumor suppressor gene; however, the precise underlying mechanisms have not been well elucidated. In the present study, we discovered that overexpression of NAG-1 induced apoptosis in U87 MG, U118 MG, U251 MG, and T98G cell lines via the intrinsic mitochondrial pathway, but not in A172 and LN-229 cell lines. NAG-1 could induce the phosphorylation of PI3K/Akt and Smad2/3 in all six tested glioblastoma cell lines, except Smad3 phosphorylation in A172 and LN-229 cell lines. In fact, Smad3 expression and its phosphorylation were almost undetectable in A172 and LN-229 cells. The PI3K inhibitors promoted NAG-1-induced glioblastoma cell apoptosis, while siRNAs to Smad2 and Smad3 decreased the apoptosis rate. NAG-1 also stimulated the direct interaction between Akt and Smad3 in glioblastoma cells. Elevating the level of Smad3 restored the sensitivity to NAG-1-induced apoptosis in A172 and LN-229 cells. In conclusion, our results suggest that PI3K/Akt and Smad-dependent signaling pathways display opposing effects in NAG-1-induced glioblastoma cell apoptosis.

## Introduction

Nonsteroidal anti-inflammatory drug (NSAID) activated gene-1 (NAG-1), a divergent member of the transforming growth factor-beta (TGF-β)/bone morphogenetic protein (BMP) superfamily, was identified by PCR-based subtractive hybridization in NSAID-treated HCT-116 colorectal cancer cells [1]. Due to identified by several other research groups, NAG-1 is also designated as macrophage inhibiting cytokine 1 (MIC-1) [2], placental transformation growth factor beta (PTGFB) [3], prostate derived factor (PDF) [4], placental bone morphogenetic protein (PLAB) [5], growth differentiation factor-15 (GDF-15) [6], and PL74 [7].

The human NAG-1 locus has been mapped to 19p12.1-13.1 and the NAG-1 protein is encoded by two exons [3]. After dimerization of the full length pro-NAG-1 precursor by a specific disulfide linkage, the dimeric pro-protein undergoes proteolytic cleavage catalyzed by furin-like protease at the amino acid target sequence RXXR resulting in the release of a 112 amino acid C-terminal dimeric mature region. The mature dimer is then secreted into the extracellular media [8] [9]. A variety of signaling pathways may contribute to the stringent regulation of NAG-1 expression, secretion, and stromal storage [8] [9].

NAG-1 plays remarkable multifunctional roles in controlling diverse physiological and pathological processes. The functions mediated by secreted NAG-1 include the control of embryonic, osteogenic, and hematopoietic development, the regulation of immune response, cartilage and bone formation, and adipose tissue function, the participation in the cellular stress, inflammation, and the process of tissue injury and repair [8] [9]. NAG-1 also plays important roles in the development and progression of cancer [8] [9] [10]. NAG-1 expression is markedly increased in melanoma and gastrointestinal, prostate, pancreatic, colorectal, breast, and thyroid cancer [11]. NAG-1 has been described as 1 of the 20 best cancer biomarkers based on transcriptional profiling of a broad range of mainly epithelial tumor types, including renal cell carcinoma, adenocarcinoma of the colon, ovary and esophagus and also in melanoma [12]. Aberrant increases in the serum levels of secreted NAG-1 correlate with poor prognosis and patient survival rates in some cancers. Measurement of the secreted form of NAG-1 has been proposed as a marker for cancer progression and risk assessment [13] [14] [15].

Glioblastoma multiforme (GBM) is a grade IV astrocytoma with a median survival of 12 months despite current multi-modal treatment options [16]. Although NAG-1 expression is enhanced in many cancers, while in contrast, it has been reported that NAG-1 expression in glioblastoma cell lines is significantly lower than in benign glioma cells and normal human astrocytes [17]. Strelau also reported that primary glioblastoma have less NAG-1 expression compared to other gliomas [18]. NAG-1 can induce cell cycle arrest and apoptosis in several cancer cell lines [9] [19], but the proapoptotic role and the underlying mechanisms of NAG-1 in gliomas have not been well elucidated. Here we report that PI3K/Akt and Smad-dependent signaling pathways possess opposing effects in NAG-1-induced glioblastoma cell apoptosis.

## Materials and Methods

### Cell culture and reagents

The human glioblastoma cell lines U87 MG, U118 MG, U251 MG, A172, LN-229, and T98G were purchased from Shanghai Institute of Cell Biology, Chinese Academy of Sciences (Shanghai, China). The cells were cultured in DMEM supplemented with 10% fetal bovine serum (Gibco, Grand Island, NY, USA).

The antibodies against NAG-1, Bcl-2, Bax, caspase-3, caspase-8, caspase-9, cytochrome c, p-PI3K(p85 Tyr458)/PI3K(p85), p-Akt(Ser473)/Akt, p-ERK1/2(Thr202/Tyr204)/ERK1/2, p-Smad2(Ser465/467)/Smad2, p-Smad3(Ser423/425)/Smad3, and β-actin were purchased from Cell Signaling Technologies (Beverly, MA, USA). Wortmannin, LY294002, Smad2 siRNA, and Smad3 siRNA were purchased from Santa Cruz Biotechnology (Santa Cruz, CA, USA). Ac-IETD-FMK and Ac-LEHD-FMK were purchased from KeyGEN Biotech Co., Ltd. (Nanjing, China). Enhanced chemiluminescence (ECL) detection system was purchased from Amersham Life Science (Arlington Heights, Illinois, USA). Human NAG-1 ELISA Kit was obtained from Huamei Biological Company (Wuhan, China). Mitochondrial membrane potential assay kit with JC-1 and protein A/G agarose beads were obtained from Beyotime Institute of Biotechnology (Shanghai, China). X-treme GENE siRNA transfection reagent was purchased from Roche Applied Science.

### Adenovirus infection

NAG-1-expressing, Smad3-expressing, and control adenovirus vectors (designated as Ad-NAG-1, Ad-Smad3, and Ad-Con) were purchased from Benyuan Zhengyang Gene Technology Co., Ltd. (Beijing, China). Cells were seeded in 60 mm dishes. After infection, the cells were incubated with serum free media for different time as indicated.

### Western blot analysis

60 µg of total protein extract was resolved by SDS-PAGE and transferred to nitrocellulose membranes. The membranes were blocked in 5% milk and probed with primary antibodies overnight at 4°C. Bound antibody was detected with the secondary antibody and the ECL detection system according to the manufacturer’s manual.

### ELISA assay

NAG-1 levels in the cell culture media were measured using ELISA kit according to the manufacturer’s protocol. In brief, 100 µl of each standard and sample were added into appropriate wells. After incubation and wash procedures, 100 µl of biotinylated antibody was added to each well. The incubation and wash procedures were repeated and 100 µl of streptavidin solution was added. Then after incubation and wash procedures, 100 µl of TMB one-step substrate reagent was added and incubated in the dark. Finally, 50 µl of stop solution was added to each well. Absorbance at 450 nm was read immediately using a microplate reader.

### Apoptosis analysis by flow cytometry

The percentage of apoptotic cells was analyzed using flow cytometry (FCM). Cells were harvested and washed with PBS. Cell apoptosis was measured using two-color analysis of fluoresce in isothiocyanate-labeled annexin V binding and propidium iodide (PI) uptake with Becton Dickinson fluorescence-activated cell sorter (FACS) apparatus.

### Mitochondrial membrane potential (ΔΨ_m_) assay

Changes in the mitochondrial membrane potential were determined using the fluorescent lipophilic cationic probe JC-1 as described previously [20]. JC-1 accumulates in the mitochondria in proportion to ΔΨ_m_, forming aggregates that fluoresce red. In the cytoplasm, JC-1 exists as monomers that fluoresce green. The ratio of red fluorescence to green fluorescence was used as a surrogate for ΔΨ_m_. After 30 mins incubation with 5 µM JC-1 at dark in a 5% CO_2_ atmosphere at 37°C incubator, red fluorescence (excitation, 570 nm; emission, 595 nm) and green fluorescence (excitation, 485 nm; emission, 535 nm) were measured using a spectrofluorimeter.

### RNA interference

Smad2 siRNA, Smad3 siRNA, and control siRNA products were transfected into glioblastoma cells using X-treme GENE siRNA transfection reagent according to the manufacturer’s instruction. Specific knockdown of Smad2 and Smad3 was verified by western blot analysis.

### Co-immunoprecipitation assay

Aliquots containing 400 µg of protein were cleared with 10 µl protein A/G agarose beads. Akt or Smad3 protein was immunoprecipitated from the whole cell lysates using anti-Akt or anti-Smad3 antibody after incubation for 8 h followed by the addition of 20 µl protein A/G agarose beads and continued incubation overnight at 4°C. Immunoprecipitates were washed, and subsequently subjected to western blot analysis using anti-Smad3 or anti-Akt antibody.

### Statistical analysis

Statistical analysis was performed with SPSS software (version 10.0; SPSS, Chicago, IL). All the data were presented as the mean ± standard error of the mean (SEM). The difference between groups was analyzed by one-way analysis of variance (ANOVA). Statistical significance was defined as *P*<0.05.

## Results

### NAG-1-induced apoptosis in a restricted set of glioblastoma cell lines

**Figure 1:**
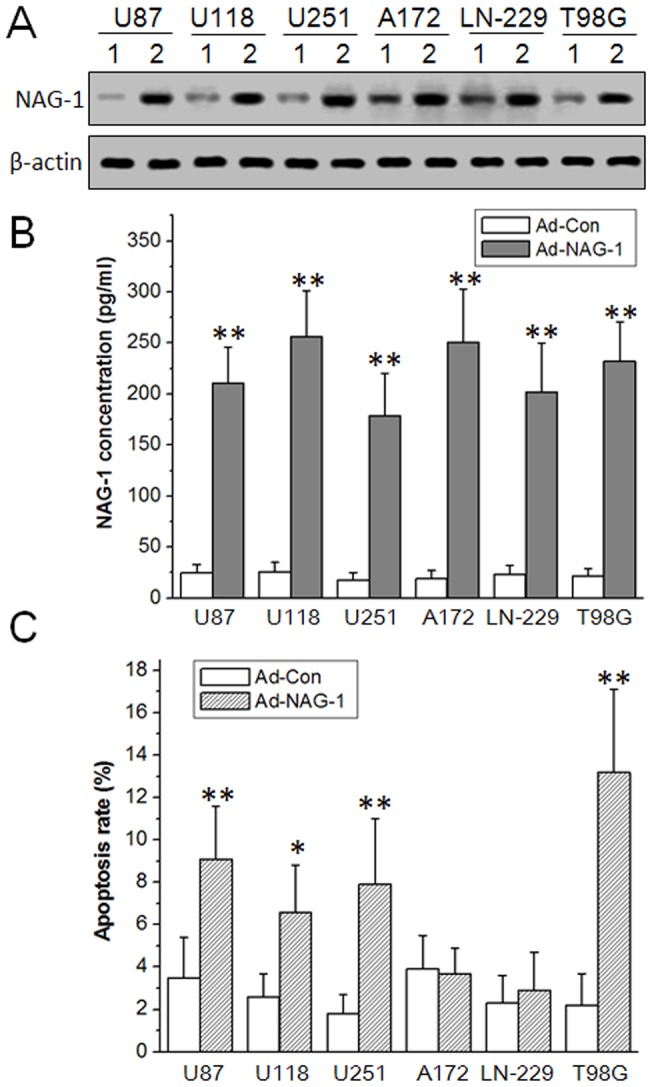
NAG-1-induced apoptosis in a restricted set of glioblastoma cell lines. U87 MG, U118 MG, U251 MG, A172, LN-229, and T98G glioblastoma cell lines were infected by Ad-NAG-1 and Ad-Con at 50 MOI (multiplicity of infection), respectively. A, 24 h after infection, NAG-1 overexpression in cell lysates was verified by western blot. B, 24 h after infection, secreted NAG-1 in culture media was determined by ELISA. C, 48 h after infection, cell apoptosis was analyzed by FCM. 1, Ad-Con, 2, Ad-NAG-1. *, *P*<0.05, **, *P*<0.01 versus Ad-Con.

NAG-1 may influence the proliferation, differentiation, migration, invasion, survival, and apoptosis of cancer cells, but there is much contradictory evidence with regard to the role of NAG-1 in cancer cells [9]. Some experimental results suggest that NAG-1 has anti-tumorigenic properties, while other experimental results suggest that it has pro-tumorigenic properties [19]. To confirm whether NAG-1 induces glioblastoma cell apoptosis, NAG-1-expressing adenovirus vector was constructed and glioblastoma cells infected by adenoviruses were analyzed by FCM. As shown in Fig. 1 A and B, NAG-1 protein levels in cell lysates and secreted NAG-1 protein levels in growth media increased significantly as determined by western blot and ELISA. FCM results showed that NAG-1 overexpression significantly increased the proportion of apoptotic cells in U87 MG, U118 MG, U251 MG, and T98G cell lines, but not in A172 and LN-229 cell lines (Fig. 1 C). These results demonstrate that NAG-1 can induce apoptosis in a restricted set of glioblastoma cell lines.

### NAG-1-induced glioblastoma cell apoptosis via a mitochondrial pathway

**Figure 2:**
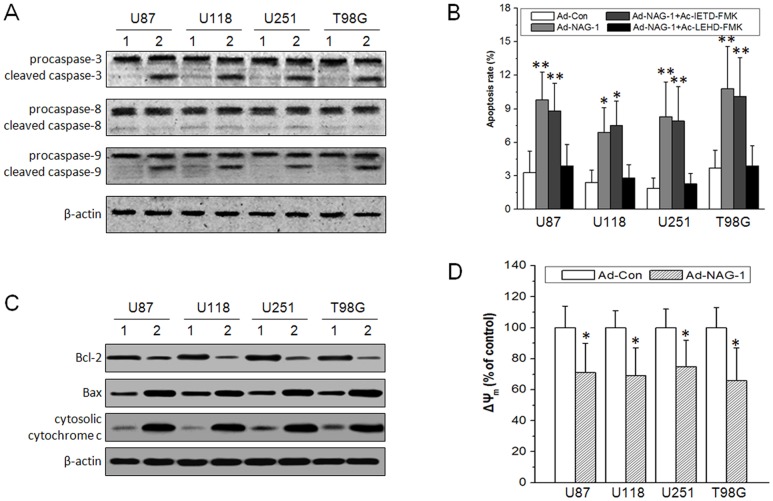
NAG-1-induced glioblastoma cell apoptosis via the intrinsic mitochondrial pathway. U87 MG, U118 MG, U251 MG, and T98G glioblastoma cell lines were infected by Ad-NAG-1 and Ad-Con at 50 MOI, respectively. 48 h after infection, the characteristics of NAG-1-induced apoptosis were analyzed. A, cleavage of caspase-3, caspase-8, and caspase-9 as determined by western blot. B, at the same time of infection, 50 µM Ac-IETD-FMK or Ac-LEHD-FMK was added into the culture media, and cell apoptosis was analyzed by FCM. C, Bcl-2, Bax, and cytosolic cytochrome c as determined by western blot. D, the mitochondrial membrane potential (ΔΨ_m_) was detected by JC-1 staining. 1, Ad-Con, 2, Ad-NAG-1. *, *P*<0.05, **, *P*<0.01 versus Ad-Con.

In mammalian cells, apoptosis can be triggered by the extrinsic pathway stimulated by the death receptor and the intrinsic pathway regulated by Bcl-2 family members in the mitochondrion [21]. To characterize the apoptosis pathways activated by NAG-1 overexpression, firstly, proteolytic cleavage of caspase-3, caspase-8, and caspase-9 was analyzed by western blot. As shown in Fig. 2 A, cleaved caspase-3 and cleaved caspase-9 increased significantly, but cleaved caspase-8 was undetectable. Next, the role of caspase-8 and caspase-9 in NAG-1-induced apoptosis was analyzed using caspase-8 inhibitor Ac-IETD-FMK and caspase-9 inhibitor Ac-LEHD-FMK. As shown in Fig. 2 B, NAG-1-induced apoptosis was abolished by Ac-LEHD-FMK, but not by Ac-IETD-FMK. Thus, NAG-1-induced glioblastoma cell apoptosis appear to be mediated by the intrinsic pathway. A limiting step in the intrinsic apoptotic pathway is the damage of mitochondria and the release of cytochrome c from mitochondria into the cytosol. The members of Bcl-2 family are essential for regulating the mitochondrial integrity, and the increase in mitochondrial permeability transition is accompanied by a collapse in mitochondrial membrane potential (ΔΨ_m_) [20]. As shown in Fig. 2 C, NAG-1 overexpression caused decreased Bcl-2 expression, increased Bax expression, and elevated level of cytosolic cytochrome c in glioblastoma cells. NAG-1-induced decline in ΔΨ_m_ was also detected (Fig. 2 D). These results indicate that NAG-1 can induce glioblastoma cell apoptosis via a mitochondrial pathway.

### NAG-1-activated signaling pathways in glioblastoma cells

**Figure 3:**
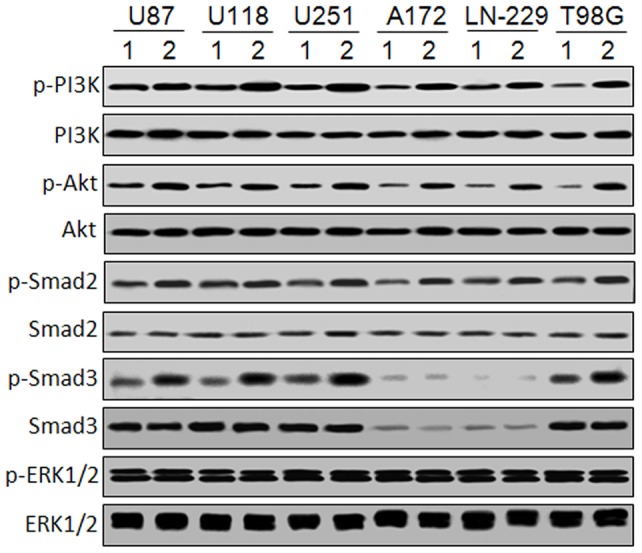
NAG-1-activated signaling pathways in glioblastoma cells. U87 MG, U118 MG, U251 MG, A172, LN-229, and T98G glioblastoma cell lines were infected by Ad-NAG-1 and Ad-Con at 50 MOI, respectively. 24 h after infection, phosphorylation of PI3K(p85 Tyr458), Akt(Ser473), Smad2(Ser465/467), Smad3(Ser423/425), and ERK1/2(Thr202/Tyr204) was analyzed by western blot.

The receptor and the signaling pathways of NAG-1 remain uncertain, although several of its biological activities have already been described. It has been reported that the downstream signaling cascades activated by NAG-1 include PI3K/Akt, Smad2/3, and MAPK/ERK1/2 [8] [9]. To test whether these signaling cascades are activated by NAG-1 in glioblastoma cells, phosphorylation of PI3K/Akt, Smad2/3, and ERK1/2 was analyzed by western blot. As shown in Fig. 3, a prominent induction of PI3K/Akt and Smad2 phosphorylation was detected in all six glioblastoma cell lines. Smad3 phosphorylation was induced in U87 MG, U118 MG, U251 MG, and T98G cell lines, while Smad3 and its phosphorylation were almost undetectable in A172 and LN-229 cell lines. ERK1/2 phosphorylation was not induced in all tested cell lines. These results suggest that PI3K/Akt and Smad2/3 signaling cascades may be involved in NAG-1-induced glioblastoma cell apoptosis.

### Involvement of PI3K/Akt and Smad2/3 in NAG-1-induced glioblastoma cell apoptosis

**Figure 4:**
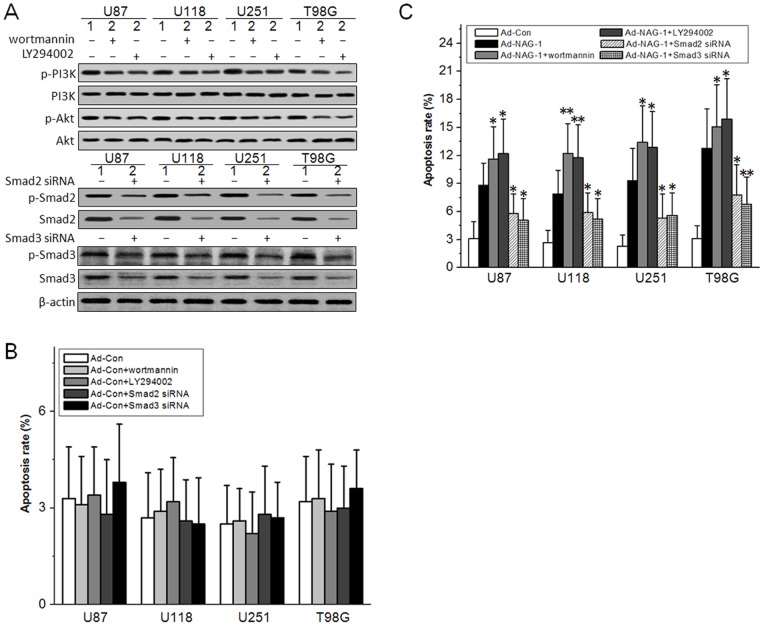
Involvement of PI3K/Akt and Smad2/3 in NAG-1-induced glioblastoma cell apoptosis. U87 MG, U118 MG, U251 MG, and T98G glioblastoma cell lines were infected by Ad-NAG-1 and Ad-Con at 50 MOI, respectively. A, at the same time of infection, 1 µM wortmannin or 10 µM LY294002 was added into the culture media. 24 h later, the Akt phosphorylation was analyzed by western blot. Smad2 or Smad3 siRNA was transfected into the cells. 24 h later, the interference effects were verified by western blot. B, C, the effect of wortmannin, LY294002, Smad2 siRNA, or Smad3 siRNA on Ad-Con or Ad-NAG-1-induced apoptosis was analyzed by FCM. 1, Ad-Con, 2, Ad-NAG-1. *, *P*<0.05, **, *P*<0.01 versus Ad-NAG-1.

PI3K/Akt pathway is a key regulator of cell growth and survival in many cancers including glioblastomas [22]. Smad3 plays a critical role in TGF-β-mediated growth inhibition and apoptosis [23]. To confirm the role of PI3K/Akt and Smads in NAG-1-induced glioblastoma cell apoptosis, the PI3K inhibitors and siRNAs to Smad2 and Smad3 were used to inhibit PI3K/Akt and Smad2/3 signaling cascades. As shown in Fig. 4 A, increased PI3K and Akt phosphorylation induced by NAG-1 overexpression was abolished by wortmannin and LY294002, and the expression level of Smad2 and Smad3 was significantly decreased by siRNAs. Then, the effects of PI3K inhibitors and siRNAs on NAG-1-induced apoptosis were analyzed by FCM. As shown in Fig. 4 C, the apoptosis rate was increased by wortmannin and LY294002, while it was decreased by siRNAs to Smad2 and Smad3. The PI3K inhibitors and Smad2/3 siRNAs had no proapoptotic effects on Ad-Con infected glioblastoma cells (Fig. 4 B). These results demonstrate that PI3K/Akt and Smad2/3 signaling cascades are involved in NAG-1-induced glioblastoma cell apoptosis.

### Inhibition of Smad3 phosphorylation by Akt in NAG-1-overexpressed glioblastoma cells

**Figure 5:**
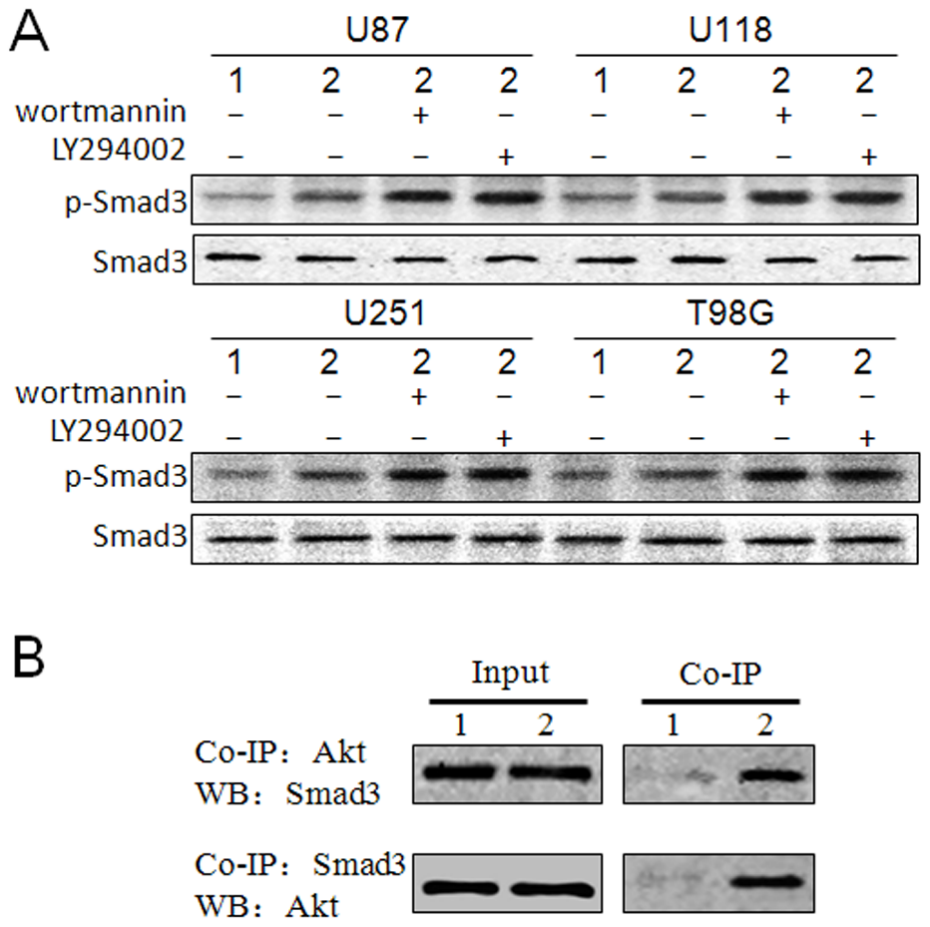
The direct interaction between Akt and Smad3 in NAG-1-overexpressed glioblastoma cells. A, U87 MG, U118 MG, U251 MG, and T98G glioblastoma cell lines were infected by Ad-NAG-1 and Ad-Con at 50 MOI, respectively. At the same time of infection, 1 µM wortmannin or 10 µM LY294002 was added into the culture media. 24 h later, the Smad3 phosphorylation was analyzed by western blot. B, U251 MG cells were infected by Ad-NAG-1 and Ad-Con at 50 MOI, respectively. 24 h later, the interaction of Akt and Smad3 was confirmed by co-immunoprecipitation. 1, Ad-Con, 2, Ad-NAG-1. Co-IP, co-immunoprecipitation. WB, western blot.

It has been reported that Akt can modulate the TGF-β signaling pathway through the physical interaction of Akt and Smad3. Akt when bound to Smad3 inhibits Smad3 phosphorylation and Smad3-induced apoptosis in hepatoma cells [24] [25]. To test whether Akt can suppress the phosphorylation of Smad3 in NAG-1-overexpressed glioblastoma cells, the PI3K inhibitors wortmannin and LY294002 were used to inhibit the Akt phosphorylation, and then the Smad3 phosphorylation was analyzed by western blot. As shown in Fig. 5 A, the level of Smad3 phosphorylation was increased by wortmannin and LY294002. The direct interaction of Akt and Smad3 was also verified in U251 MG cells infected by NAG-1-expressing adenovirus using co-immunoprecipitation (Fig. 5 B). These results suggest that PI3K/Akt inhibitors may promote NAG-1-induced glioblastoma cell apoptosis by inhibiting the interaction between Akt and Smad3.

### Restored apoptosis sensitivity in A172 and LN-229 glioblastoma cell lines

**Figure 6:**
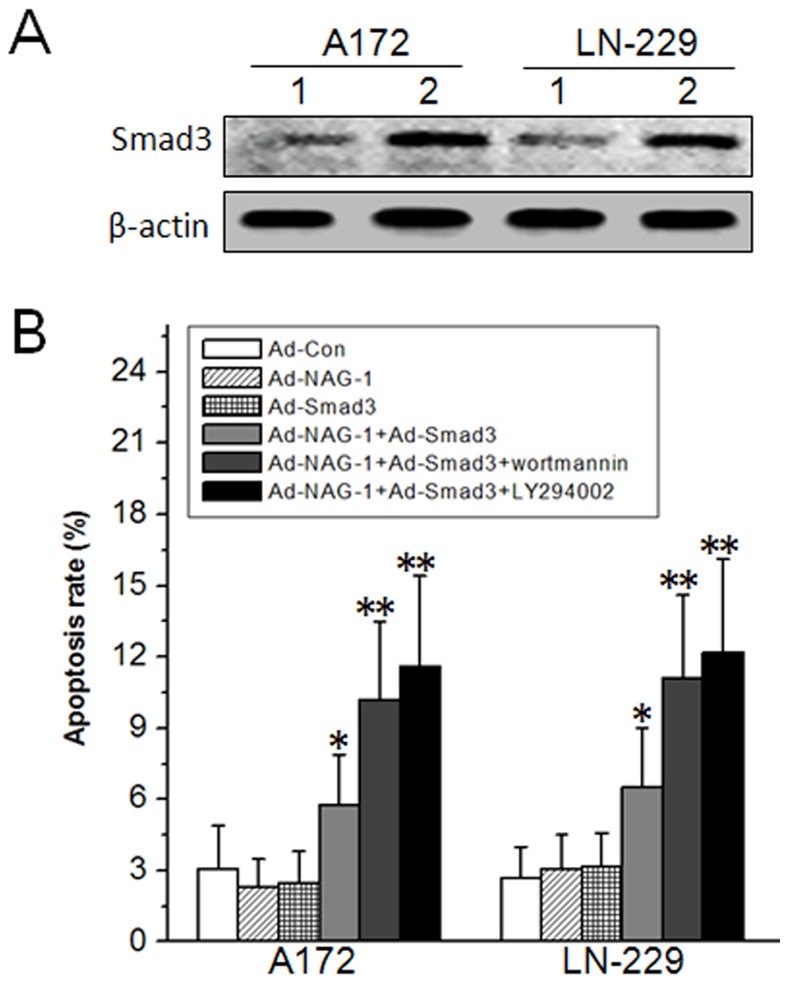
Restored apoptosis sensitivity in A172 and LN-229 glioblastoma cell lines. A, A172 and LN-229 cells were infected by Ad-Smad3 at 50 MOI. 24 h later, overexpression of Smad3 was analyzed by western blot. B, Ad-Smad3 and Ad-NAG-1 with or without wortmannin or LY294002 induced A172 and LN-229 cell apoptosis as determined by FCM after treatment for 48 h. 1, Ad-Con, 2, Ad-Smad3. *, *P*<0.05, **, *P*<0.01 versus Ad-Con.

It has been reported that the escape from TGF-β-mediated growth inhibition in malignant glioma cells is due to abnormalities in the TGF-β signaling pathway [26] [27]. As demonstrated above, NAG-1 overexpression failed to induce apoptosis in A172 and LN-229 cell lines with almost undetectable Smad3 and its phosphorylation, while siRNA to Smad3 decreased NAG-1-induced apoptosis in U87 MG, U118 MG, U251 MG, and T98G cell lines (Fig. 1 C, Fig. 3, Fig. 4 B). Thus, we speculated that elevating the level of Smad3 may restore the sensitivity to NAG-1-induced apoptosis in A172 and LN-229 cell lines. Overexpression of Smad3 was verified by western blot (Fig. 6 A). As shown in Fig. 6 B, FCM results confirmed our speculation. The PI3K inhibitors wortmannin and LY294002 further increased the apoptosis rate in A172 and LN-229 cell lines. These findings suggest that lower levels of Smad3 may be responsible for apoptosis resistance to NAG-1 in some glioblastoma cell lines.

## Discussion

Like other TGF-β family members, NAG-1 can play dual roles during cancer development and progression by negatively or positively modulating cancer cell behaviors [8] [9]. Both the anti-tumorigenic and pro-tumorigenic activity of NAG-1 is supported by experimental evidence. Therefore, the anti-tumorigenic and pro-tumorigenic properties of NAG-1 appear to be dependent on cancer cell type and context [8] [9] [10].

It has been reported that NAG-1 expression in glioblastoma cell lines is significantly lower than in benign glioma cells and normal human astrocytes, and that primary glioblastoma have less NAG-1 expression compared to other gliomas [17] [18]. Thus, NAG-1 basal expression appears to inversely correlate with tumor grade in glioma. NAG-1 overexpression inhibits the colony-forming capacity and induces apoptosis in glioblastoma cells [28]. Increased expression of NAG-1 induced by histone deacetylase inhibitor trichostatin A (TSA) may mediate, in part, TSA-induced apoptosis [17]. In the present study, we discovered that NAG-1 overexpression could induce apoptosis in U87 MG, U118 MG, U251 MG, and T98G cell lines, but not in A172 and LN-229 cell lines (Fig. 1). These findings support the hypothesis that NAG-1 appears to act as a tumor suppressor gene in glioblastomas. It also has been reported that recombinant NAG-1 has no impact on apoptosis in several glioblastoma cell lines, which might be caused by cell culture conditions such as the use of serum-free or serum-containing media [18] [28].

Apoptosis follows two main pathways, the extrinsic pathway initiated by binding of ligand of specific death receptor and the intrinsic pathway initiated at mitochondria [20]. Caspase-3, the major effector caspase, is activated by upstream effector proteins including caspase-8 and caspase-9, the apical proteases in the extrinsic and intrinsic pathways, respectively [21]. In the present study, we found that cleaved caspase-3 and caspase-9 increased after NAG-1 overexpression, and that NAG-1-induced apoptosis was abolished by Ac-LEHD-FMK (Fig. 2 A and B). The release of cytochrome c and the change in ΔΨ_m_ are the key events in intrinsic pathway of apoptosis. Bcl-2 protein family plays an important role in the regulation of mitochondrial apoptosis pathway [29]. NAG-1 overexpression also caused decreased Bcl-2 expression, increased Bax expression, elevated level of cytosolic cytochrome c, and decline of ΔΨ_m_ (Fig. 2 C and D). Collectively, our data discloses that mitochondrial apoptosis pathway is involved in NAG-1-induced glioblastoma cell apoptosis.

The specific receptors activated by secreted NAG-1 have not been precisely identified. It has been suggested that NAG-1 can mediate certain cellular responses via the stimulation of TGF-β receptors type I and II and intracellular Smad signal transduction protein complexes [8]. Overexpression of NAG-1 activates ERK1/2 and Akt signaling cascades in breast and gastric cancer cells [30]. NAG-1 can also activate PI3K/Akt, ERK1/2, and SMAD2/3 signaling pathways in cardiovascular stress responses against different stimuli [31]. Here we reported that NAG-1 overexpression could activate PI3K/Akt and Smad2/3 signaling cascades in glioblastoma cells (Fig. 3), and that NAG-1-induced apoptosis was enhanced by PI3K inhibitors and decreased by siRNAs to Smad2 and Smad3 (Fig. 4). Akt can directly interact with and sequesters unphosphorylated Smad3 at the cell membrane and in the cytoplasm, suggesting that Akt can promote survival in a kinase-independent manner [24] [25]. In the present study, we also found the direct interaction between Akt and Smad3 and elevated levels of NAG-1-induced Smad3 phosphorylation by PI3K inhibitors in glioblastoma cells (Fig. 5). These findings demonstrate that PI3K/Akt and Smad2/3 signaling cascades display opposing effects in NAG-1-induced glioblastoma cell apoptosis.

Glioma cells and other cancer cells can escape from the TGF-β anti-proliferative response by acquiring inactivating mutations in several components of the TGF-β pathway [26] [27] [32]. It has been reported that NAG-1-induced growth inhibition is abolished in TGF-β receptor type I mutant R1B/L17 cells, TGF-β receptor type II mutant RKO colon carcinoma cells, and Smad4 null MDA-MB468 breast cancer cells [33]. In the present study, we found that Smad3 and its phosphorylation were almost undetectable in A172 and LN-229 cells (Fig. 3). Smad3 is critical to inducing TGF-β-mediated apoptosis. Accordingly, the loss of Smad3 function could allow for apoptosis resistance [23]. As shown in Fig. 6, Smad3 overexpression restored the apoptosis sensitivity to NAG-1 in A172 and LN-229 cells.

In conclusion, the present study demonstrates that NAG-1 can induce apoptosis in a restricted set of glioblastoma cell lines via the mitochondrial pathway. PI3K/Akt and Smad2/3 signaling cascades possess opposing effects in NAG-1-induced glioblastoma cell apoptosis. Lower levels of Smad3 may lead to the loss of apoptosis sensitivity in response to NAG-1 in some glioblastoma cell lines.
