# Correction: Opposing Effects of PI3K/Akt and Smad-Dependent Signaling Pathways in NAG-1-Induced Glioblastoma Cell Apoptosis

**DOI:** 10.1371/journal.pone.0205391

**Published:** 2018-10-03

**Authors:** Zhiguo Zhang, Lin Wu, Julei Wang, Gang Li, Dayun Feng, Bin Zhang, Lihong Li, Jiandong Yang, Lianting Ma, Huaizhou Qin

In several instances, the methods used in this article [1] were not reported in sufficient detail to enable replication. The authors provide the following clarifications to the methods used:

- The product codes for the Smad2 siRNA, Smad3 siRNA, and control siRNA products purchased from Santa Cruz Biotechnogy are sc-38374, sc-38376 and sc-44231, respectively.
- The adenovirus infection protocol used in the study was as follows: Cells were seeded in 60 mm dishes. For adenovirus infection, serum-containing medium was replaced by serum-free medium. 50 MOI of adenoviruses were added into serum-free medium. Then cells were incubated for 1 h with frequent gentle shaking. After incubation, cells were cultured in complete medium.
- The phospho-85 antibody used in the study was Cell Signaling Technology product #4228.
- For the experiments shown in Figs 3, 4A and 5A, the authors did not include parallel blots to probe for standard housekeeping genes. This decision was made because the relative levels of phosphorylated proteins to total proteins are often used to illustrate the activity of signaling pathways, such as in [2].
- Each of the figure panels in the article represents 3 experimental replicates (n = 3).
